# 

**Published:** 1884-08

**Authors:** 


BOOKS AND PAMPHLETS RECEIVED.

*Manual of Physiology, a Text Book for Students of Medicine;* by Gerald F. Yeo, M. D., F. R. C. S. P. Blakiston, Son & Co., Philadelphia. 1884.

*The Theory and Practice of Medicine;* by Frederick T. Roberts, M. D., F. R. C. P. Fifth American edition. P. Blakiston, Son & Co., Philadelphia, 1884.

*Hooper's Physician's Vade Mecum;* vol. ii, being part of Wood's Library of Standard Medical Authors for 1884. To be had of W. T. Keener, Chicago.

*Auscultation Percussion and Urinalysis;* by C. Henri Leonard. Illustrated Medical Journal Co., Detroit. 1884.

*Student's Manual of Electro-Therapeutics;* by R. W. Amidon. Putnam's. 1884.

*Borderland Psychiatric Records—Brodromal Symptoms of Psychical Impairment;* by C. H. Hughes, M. D.

*Construction of Hospitals for the Insane;* by J. C. Spray, M. D.

*The New York State Medical Association Report.*

*Report of Cases of Ovariotomy;* by W. H. Kane King, M. D.

*Peroxide of Hydrogen in Suppurative Conjunctivitis;* by A. E. Prince, M. D.

*Peroxide of Hydrogen in Diphtheria;* by R. J. Nunn, M. D.

*Medical Annals of Baltimore;* by John R. Quinan.

*Prospectus of a New Work on Physiology;* by W. H. Wriplett, M. D.

*Some Recent Theories Regarding the Pathogeny of Sympathetic Ophthalmia;* by Samuel Theobold, M. D.

*Twenty-Third Annual Report of the Cincinnati Hospital.*

*Memoria Cientifico—Descriptiva de las aquas Minero—Medicinales de la Favorita de Cabana.* Madrid. 1884.

*Criminal Responsibility of the Insane;* by O. Evarts, M. D.

*Annual Address before the Medical Society of the State of California;* by Ira E. Oatman, M. D.

*Paroxysmal Fever not Malarial;* by J. H. Musser, M. D.

*Sixth Annual Announcement of the Central College of Physicians and Surgeons.* Indianapolis, Ind.

*Quarantine and Sanitary Operations of the Board of Health of the State of Louisiana;* by Joseph Jones, M. D.

